# Supplementary material for: DMC1 stabilizes crossovers at high and low temperatures during wheat meiosis
Source: Front Plant Sci. 2023 Aug 8;14:1208285. doi: 10.3389/fpls.2023.1208285 (PMC10442654; doi:10.3389/fpls.2023.1208285)
Supplement: Supplementary file 1 [file Presentation_1.zip › Supplementary Figure 1.pptx]

## Slide 1
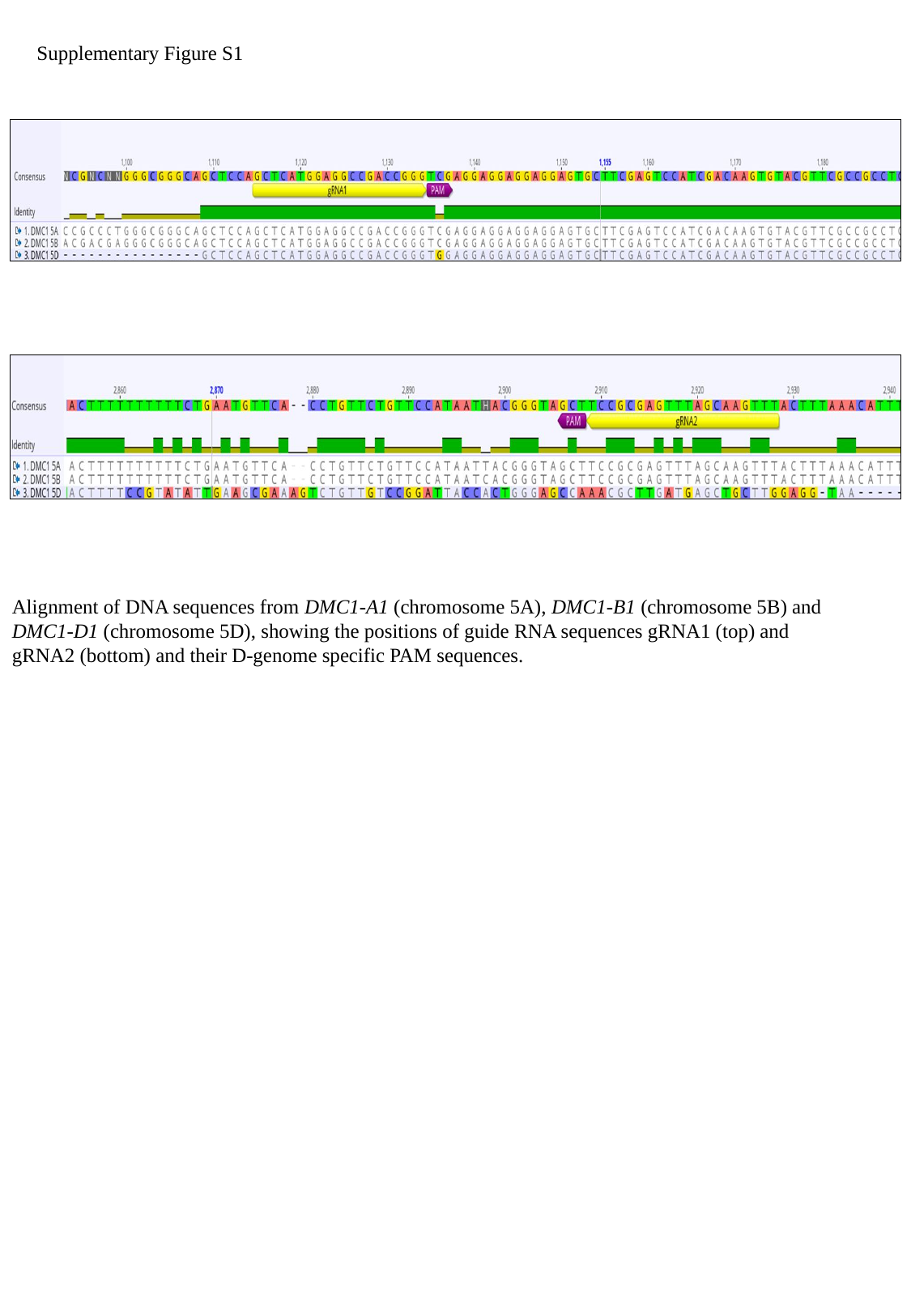

Supplementary Figure S1
Alignment of DNA sequences from DMC1-A1 (chromosome 5A), DMC1-B1 (chromosome 5B) and DMC1-D1 (chromosome 5D), showing the positions of guide RNA sequences gRNA1 (top) and gRNA2 (bottom) and their D-genome specific PAM sequences.
