# Supplementary material for: DMC1 stabilizes crossovers at high and low temperatures during wheat meiosis
Source: Front Plant Sci. 2023 Aug 8;14:1208285. doi: 10.3389/fpls.2023.1208285 (PMC10442654; doi:10.3389/fpls.2023.1208285)
Supplement: Supplementary file 1 [file Presentation_1.zip › Supplementary Figure 2.pptx]

## Slide 1
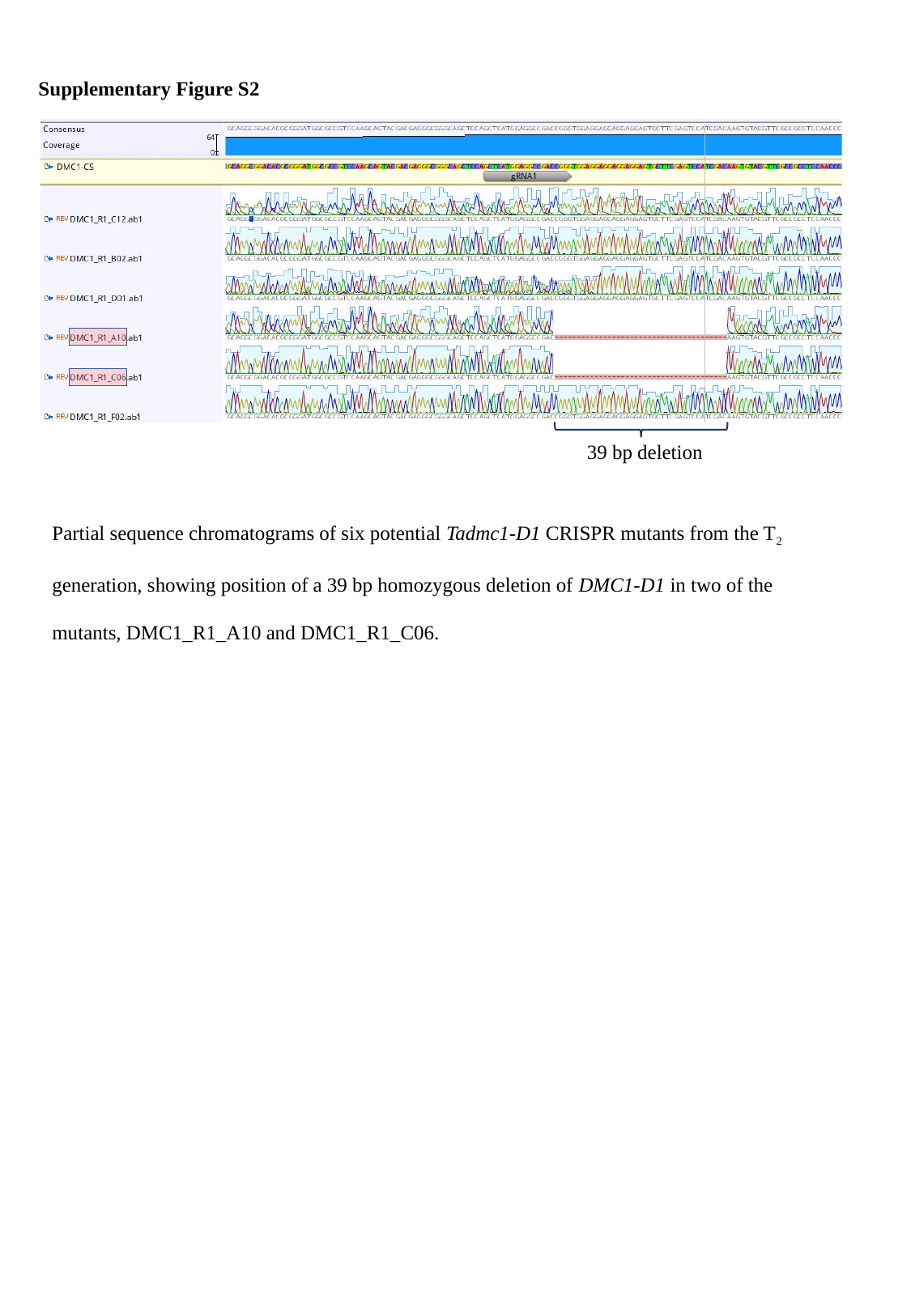

Supplementary Figure S2
39 bp deletion
Partial sequence chromatograms of six potential Tadmc1-D1 CRISPR mutants from the T2 generation, showing position of a 39 bp homozygous deletion of DMC1-D1 in two of the mutants, DMC1_R1_A10 and DMC1_R1_C06.
